# COVID-19 Drug Development

**DOI:** 10.4014/jmb.2110.10029

**Published:** 2021-12-01

**Authors:** Seungtaek Kim

**Affiliations:** Zoonotic Virus Laboratory, Institut Pasteur Korea, Seongnam 13488, Republic of Korea

**Keywords:** COVID-19, SARS-CoV-2, drug repurposing, monoclonal antibody, convalescent plasma

## Abstract

Diagnostics, vaccines, and drugs are indispensable tools and control measures employed to overcome infectious diseases such as coronavirus disease 2019 (COVID-19). Diagnostic tools based on RT-PCR were developed early in the COVID-19 pandemic and were urgently required for quarantine (testing, tracing and isolation). Vaccines such as mRNA vaccines and virus-vectored vaccines were also successfully developed using new platform technologies within one year after identifying severe acute respiratory syndrome coronavirus 2 (SARS-CoV-2) as the causative agent of COVID-19. Drug development has been conducted in various ways including drug repurposing, convalescent plasma therapy, and monoclonal antibody development. Among the above efforts, this review examines COVID-19 drug development along with the related and upcoming challenges.

## Introduction

Coronavirus disease 2019 (COVID-19) is a novel infectious disease caused by severe acute respiratory syndrome coronavirus 2 (SARS-CoV-2) [1]. As of October 19, 2021, more than 240 million cases of COVID-19 were reported and almost 5 million people have died of this infectious disease [2]. Although most COVID-19 patients do not experience symptoms or show only mild disease, some (~ 20%) experience severe disease and may die [3]. Advanced age and underlying medical conditions are known risk factors for severe COVID-19.

SARS-CoV-2 is very similar to SARS-CoV, which was identified in China in 2002~2003, with both viruses showing approximately 80% resemblance in their viral genome sequences [1]. Like other coronaviruses, SARS-CoV-2 is an enveloped, spherical virus and contains a positive-sense, single-stranded RNA as its genome. Thus, all steps of the viral life cycle occur within the cytoplasm of infected host cells. The first step of the SARS-CoV-2 life cycle is entry of the virus into the host cell, which is mediated by the interactions between viral spike glycoprotein and ACE2 (angiotensin-converting enzyme 2), a host receptor protein [1]. Importantly, the presence of ACE2 on the plasma membrane is a major determinant of host tropism in SARS-CoV-2 infection. In this entry process, another protein, TMPRSS2 (transmembrane serine protease 2), is also involved by facilitating the fusion between viral membrane and host cell membrane, which is required to release the viral genome into the cytoplasm [4]. This fusion occurs following the cleavage of viral spike protein by TMPRSS2 serine protease activity. Once the virus enters the cell, the viral genomic RNA is released and used for translation of viral proteins and also for transcription/replication of viral RNA. Viral particle assembly occurs in the ERGIC (ER-Golgi intermediate compartment) and assembled particles are secreted out of the cells [5].

While there are numerous viruses belonging to the *Coronaviridae* family, seven coronaviruses are known to infect humans (*i.e.*, 229E, OC43, SARS-CoV, NL63, HKU1, MERS-CoV (Middle East respiratory syndrome coronavirus) and SARS-CoV-2) and they were identified after the year 2000 except for 229E and OC43, which were reported in the 1960s. Among these human coronaviruses, only three coronaviruses (SARS-CoV, MERS-CoV, and SARS-CoV-2) are known to cause severe respiratory diseases and were reported in 2002, 2012, and 2019, respectively. The recent, frequent emergence of these highly pathogenic coronaviruses emphasizes the need for concerted global efforts to better prepare for these recurring challenges.

The lung is a main target of SARS-CoV-2 infection and the incubation period between virus exposure and symptom onset is approximately 4 ~ 5 days, although it varies between 2 and 14 days. The viral load increases as the virus replication continues until the immune response of the infected person starts. In most cases, the viral load decreases as a result of the immune response and the infected person recovers spontaneously without therapeutic intervention. However, people with high risk factors may suffer from severe COVID-19 and need treatment. Treatment of patients with COVID-19 cannot be achieved with any single drug because COVID-19 is known to be a bi-phasic (or multi-phasic) disease which needs differential therapeutic approaches [6]. The first phase of COVID-19 is the viral replication phase during which drugs that inhibit viral replication can be helpful. The second phase of COVID-19 is an inflammatory phase in which the excessive immune response plays a main role in damaging the infected individual: thus, drugs that reduce excessive immune response can be helpful. Without knowing these distinctive stages of COVID-19, drugs that are used for COVID-19 patients may not help but rather exacerbate the disease [7].

Many laboratories and companies simultaneously began developing drugs for COVID-19 very early in the pandemic and most of this drug development can be summarized in the following four categories: 1) drug repurposing (or repositioning), 2) monoclonal antibody development, 3) convalescent plasma therapy, and 4) new drug development.

## Drug Repurposing

In general, drug development requires a very long period of research and investment (typically more than 10 ~ 15 years). It usually starts from basic research and proceeds to preclinical trials (animal experiments), followed by phase 1, 2 and 3 clinical trials (testing in humans) prior to FDA approval. Despite these efforts, the chance of successful drug development is very low (less than 10%) [8]. Therefore, this kind of traditional drug development is not practical in the current COVID-19 pandemic since we need therapeutics as soon as possible.

Drug repurposing is a rapid and effective approach to reposition drugs that have already been approved for use in other indications, for treatment of COVID-19 [8]. A main advantage of drug repurposing is that the approved drugs are already well documented in terms of drug safety, pharmacokinetics, etc., because they have passed all the steps required for FDA approvals. Thus, if antiviral efficacy against SARS-CoV-2 is demonstrated in vitro or in vivo, the drugs can be directly tested in phase 2 or 3 clinical trials without being tested in preclinical or phase 1 clinical trials, as phase 1 clinical trials are usually conducted to test drug safety in healthy people. Since the beginning of the COVID-19 pandemic, numerous laboratories have conducted experiments for drug repurposing using SARS-CoV-2 spike-bearing pseudovirus or authentic SARS-CoV-2. Of many potential drug candidates, remdesivir, lopinavir/ritonavir, and chloroquine (or hydroxychloroquine) have received more attention than any of the other drugs and were the targets of intensive investigation early in the COVID-19 pandemic. Remdesivir is a nucleoside analog and had originally been developed for treatment of Ebola virus infection but it was also being investigated as a potential MERS drug prior to the COVID-19 pandemic [9]. Lopinavir/ritonavir is an antiviral drug targeting HIV (human immunodeficiency virus) viral protease and is being used for treatment of AIDS (acquired immunodeficiency syndrome) patients. Lastly, chloroquine (or hydroxychloroquine) has been used as an anti-malaria drug. Many global clinical trials were conducted for these drugs and only remdesivir was approved by the FDA for treatment of COVID-19 patients, although the clinical efficacy of remdesivir is still controversial [10-14]. The reason for this conflicting clinical outcome for remdesivir is that the drug was mainly tested for treatment of severe COVID-19 patients in which antiviral drugs may not be very useful. However, a recent result of the remdesivir clinical trial for patients with mild COVID-19 showed a higher efficacy, which demonstrates again that antiviral drugs should be used in the earlier virus replication phase of COVID-19 [15].

While remdesivir, lopinavir/ritonavir, and chloroquine (or hydroxychloroquine) were developed as drugs with antiviral mechanisms against SARS-CoV-2, others, such as dexamethasone, tocilizumab, and baricitinib, were developed as anti-inflammatory drugs. After several large-scale clinical trials, the clinical efficacy of these drugs was demonstrated, and they were approved for patients with severe COVID-19 [16-18].

Screening for drug repurposing was also conducted by many laboratories and some results were published as early as March, 2020, which was only 2 months after identification of SARS-CoV-2 as the causative agent of COVID-19 [19]. Drugs identified in these screening efforts are being tested in many clinical trials. Among numerous drug candidates identified through screening, camostat and nafamostat are notable in that both drugs are already being used for the treatment of pancreatitis in some countries, and moreover, they have a well-known mechanism of action as inhibitors of the serine protease activity of TMPRSS2 [4, 20-22].

## Monoclonal Antibody Development

Monoclonal antibodies can be developed more rapidly than small molecule inhibitors and several of them have already been successfully developed and authorized for use in patients with mild COVID-19 (Table 1) [23]. As antiviral drugs, monoclonal antibodies are effective in preventing progression to severe COVID-19 and reducing mortality for patients with mild COVID-19 [24-27] . However, these monoclonal antibodies come with caveats, for example, high price tag, limitations in large-scale production, and vulnerability to virus variants. The antiviral mechanism of monoclonal antibodies is to block virus entry by neutralizing infectious virus particles. Since neutralization is dependent on the physical interaction between antibodies and viral spike protein, the emergence of virus variants could weaken the neutralization capacity of monoclonal antibodies. This has led to combinations of different monoclonal antibodies or the development of monoclonal antibodies targeting a more conserved region of the viral spike protein. Despite the high clinical efficacy, treatment with monoclonal antibodies is mostly confined to mildly affected COVID-19 patients with high risk factors, in part due to the administration method (*i.e.*, injection).

## Convalescent Plasma Therapy

Using convalescent plasma for treatment of infectious diseases is not a new concept and it is often considered as an option when there are no other therapeutics available. Convalescent plasma is a source of neutralizing antibodies and the mechanism of action is very similar to that of monoclonal antibodies. However, unlike monoclonal antibodies, using convalescent plasma has limitations because it has to be donated from patients who recovered from COVID-19 and standardization of convalescent plasma is not easy since the neutralizing antibody titers vary depending on the different sources. While there have been some promising case reports, the result of a recent large-scale clinical trial failed to show any clinical efficacy of convalescent plasma in high-risk patients with COVID-19 [28].

## New Drug Development

**Fig. 1:**
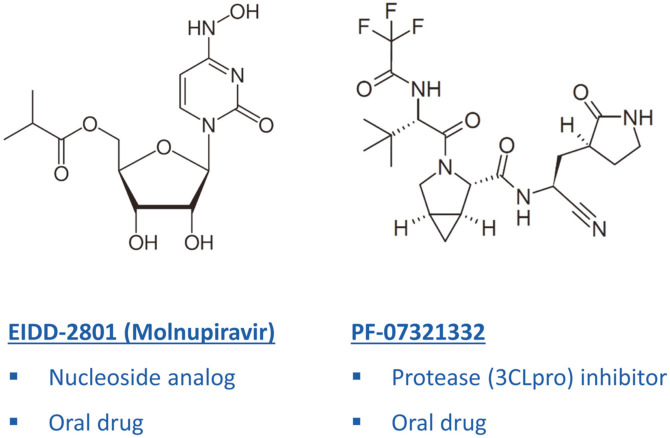
Representative new drug candidates for treatment of COVID-19. See text for more detailed explanation.

Although new drug development is not practical in a pandemic situation, some drugs are being actively developed based on their previous investigation results. Drugs being developed by Merck and Pfizer are representative examples that fall into this category (Fig. 1). Merck is developing molnupiravir (EIDD-2801), a nucleoside analog, and according to the recent interim result of the phase 3 clinical trial, COVID-19 patients who were treated with this drug showed an approximately 50% reduction in hospitalization compared to the placebo group and there was no death reported [29]. In terms of mechanism of action, remdesivir and molnupiravir are similar to each other in that both are RNA-dependent RNA polymerase inhibitors; however, they are also different in that while remdesivir is an injection drug, molnupiravir is an oral drug. Being orally available is a very important feature of antiviral drugs because if drugs can be taken orally at home without visiting hospitals at the early virus replication phase, they would be able to substantially reduce the number of hospitalizations and subsequent mortality. Molnupiravir is not completely a new drug. Prior to the COVID-19 pandemic, this drug was known to have substantial antiviral activity against numerous RNA viruses [30-33] .

On the other hand, the drug being developed by Pfizer (PF-07321332) belongs to a different class of antiviral drugs [34]. As an oral drug, PF-07321332 inhibits viral protease activity (3CL^pro^ or M^pro^). This protease activity is essential in the coronavirus life cycle since it cleaves the viral polyprotein into individual, mature proteins. Without this protease activity, the coronavirus life cycle cannot proceed and ultimately virus replication stops. PF-07321332 is also not a new drug as it was being developed during the SARS outbreak in 2002~2003, although this outbreak lasted less than 8 months. Since there are no longer reports of patients with SARS, the drug was never tested in clinical trials and therefore the SARS drug discovery program was halted. When the COVID-19 pandemic started, this program was revived because SARS-CoV-2 and SARS-CoV are similar to each other.

Being orally available is a common key feature of these two drugs and this is very important in a pandemic situation as exemplified by oseltamivir, which played a significant role in the past flu pandemic. In addition, these two drugs inhibit the two well-known antiviral targets (*i.e.*, RNA-dependent RNA polymerase and viral protease) and there have been many successful examples of HIV and HCV (hepatitis C virus) drug development targeting these viral proteins [35].

## Challenges

As discussed above, many different approaches have been taken to develop therapeutics against COVID-19. Some have been successful so far (*i.e.*, monoclonal antibodies), but others have had mixed results, showing both success and disappointment (*i.e.*, drug repurposing, convalescent plasma).

An ideal drug for treatment of COVID-19 patients should have the following features: 1) it can be taken orally, 2) it prevents progression to severe COVID-19, 3) it is affordable and easily accessible even by people in low-income countries, and 4) it works against SARS-CoV-2 variants and potentially even against related coronaviruses such as MERS-CoV and SARS-CoV.

Vaccines will play a major role in terminating the current pandemic. But the importance of drugs should not be ignored, as demonstrated by the antiviral drugs developed against HIV or HCV. Both are the causative agents of chronic infectious diseases (AIDS and chronic hepatitis C, respectively) and also causes of substantial global socioeconomic burden. Even after decades of intensive investigations, no effective vaccine has been successfully developed for these infectious diseases. However, antiviral drug development has been very successful to the extent that these diseases have become manageable. Surprisingly, we are now even expecting to see the eradication of HCV from the planet by antiviral drugs.

We have witnessed the frequent emergence of highly pathogenic coronaviruses since the year 2002, and yet more coronavirus outbreaks could potentially occur in the future due to spillover events [36]. Thus, preparing for future pandemics is not an option that we can afford any longer, and therefore, drug development for broad-spectrum antiviral agents (or at least pan-coronavirus inhibitors) should be the main focus of efforts to respond to current and future global challenges.

## Figures and Tables

**Table 1:** Approved monoclonal antibodies.

Name	Sponsor	Approval
REGEN-COV (casirivimab and imdevimab)	Regeneron	EUA (November 2020)
Bamlanivimab and etesevimab	Eli Lilly	EUA (February 2021)
Sotrovimab	GSK and Vir Biotechnology	EUA (May 2021)
Regdanvimab	Celltrion	EUA in South Korea (February 2021)
